# Knowledge and Attitudes of Saudi Emergency Physicians toward t-PA Use in Stroke

**DOI:** 10.1155/2018/3050278

**Published:** 2018-10-01

**Authors:** Ali M. Al Khathaami, Haya Aloraini, S. Almudlej, Haifa Al Issa, Nourhan Elshammaa, Sami Alsolamy

**Affiliations:** ^1^King Abdulaziz Medical City, National Guard Health Affairs, Riyadh, Saudi Arabia; ^2^College of Medicine, King Saud Bin Abdul Aziz University for Health Sciences, Riyadh, Saudi Arabia; ^3^King Khalid University Hospital, Riyadh, Saudi Arabia

## Abstract

**Background and Objectives:**

Tissue plasminogen activator (t-PA) within 4.5 hours from onset improves outcome in patients with ischemic stroke and has been recommended by several international guidelines. Since its approval in 1996, the debate among emergency physicians continues particularly around the result interpretation of the first positive randomized controlled trial, the National Institute of Neurological Disorders and Stroke (NINDS) clinical trial. This lack of consensus might negatively affect the delivery of effective stroke care. Here we aimed to assess the knowledge and attitude of Saudi emergency physicians toward t-PA use within 4.5 hours of onset in acute ischemic stroke.

**Methods:**

A web-based, self-administered, locally designed questionnaire was sent to all emergency physicians practicing in the city of Riyadh from January to September 2017.

**Results:**

Out of 450 emergency physicians, 122 from ten hospitals in Riyadh participated in the survey, with a 27% response rate. The majority of participants were men (78%), and their mean age was 40 ± 8 years. Half of the participants were board certified, and 36% were consultants. Half of the participants consider the evidence for t-PA use in stroke within 4.5 hours of stroke onset to be controversial, and 41% recommend against its use due to lack of proven efficacy (37%), the risk of hemorrhagic complications (35%), lack of stroke expertise (21%), and medicolegal liability (9%). Nearly half were willing to administer IV t-PA for ischemic stroke in collaboration with remote stroke neurology consultation if telestroke is implemented.

**Conclusion:**

Our study detected inadequate knowledge and a negative attitude among Saudi emergency physicians toward t-PA use in acute stroke. This might negatively impact patient outcome. Therefore, we recommend developing urgent strategies to improve emergency physicians' knowledge, attitudes, and beliefs in the management of acute stroke.

## 1. Introduction

The benefit of recombinant tissue-type plasminogen activator (t-PA) for acute ischemic stroke within 4.5 h after the onset of stroke symptoms is well-established [1]. Unfortunately, only a small portion of stroke patients receive this medication worldwide. For example, the national rate of thrombolysis across the United States ranges from 3% to 5% [2, 3]. This underutilization likely arises because of multiple factors, including the lack of public awareness about recognition and response to acute stroke symptoms and signs, the complexity of stroke system of care, and the slow adoption of such practice in the medical community [4].

Emergency physicians have pivotal roles in the stroke system of care. First, emergency medical services (EMS) are involved in prehospital stroke care. On-scene recognition, prenotification, bypass, and direct transport decisions for stroke patients affect arrival to stroke centers within the appropriate time window [5, 6]. Second, the rapid recognition of stroke patients eligible for thrombolysis in the emergency department, fast triage, imaging, and timely referral to the stroke team depend on the emergency physicians. Third, in rural areas with a lack of on-site stroke expertise, emergency physicians might take the lead in t-PA administration with the support of remote neurology consultation [7].

Despite the available evidence, the debate about the effectiveness of thrombolysis for acute stroke has continued among emergency physicians for the last 20 years [8]. In Saudi Arabia, many hospitals have started thrombolytic programs, but the role and the level of engagement of Saudi emergency physicians are unknown. Here we aimed to explore the knowledge, attitude, and beliefs of Saudi emergency physicians toward the use of t-PA for acute ischemic stroke.

## 2. Materials and Methods

### 2.1. Study Design, Area, and Settings

A cross-sectional study using a web-based, locally designed, self-administered questionnaire was conducted from January to September 2017 targeting all emergency physicians practicing in emergency departments in Riyadh hospitals, Saudi Arabia. A physician was included in the study if he/she is currently licensed by the Saudi commission for health specialties as an emergency physician including those in emergency residency training program.

### 2.2. Data Collection Process

The questionnaire was sent via emails and smartphones through emergancy departments' secretaries and directors. The first part of the questionnaire contained demographic data and general characteristics. We collected data on age, gender, level of qualification, job title (rank), years of experience, country of training, level of hospital designation for stroke, hospital type, the volume of stroke patients seen, and the availability of stroke expertise and clinical care pathways in the emergency department. The second part explored the knowledge and attitude toward t-PA use within 4.5 hours of stroke onset (Table 2). Informed consent was obtained from all participants in the form of an agree/disagree option at the beginning of the survey. The Institutional Review Boards of King Abdullah International Medical Research Center, Riyadh, Saudi Arabia, have approved the study.

### 2.3. Data Analysis

Data were presented as the mean ± standard deviation (SD) for continuous variables and frequency with percentages for categorical variables. Multivariable logistic regression was used to test the association between physicians' characteristics and attitude toward t-PA use in stroke within the 4.5 hours' time window. We used the question, “Do you recommend t-PA in acute ischemic stroke within 4.5 hrs of onset for eligible patients?” as a dependent variable. The model included age, gender, nationality, years of experience, working in hospital with stroke center, board certification, job rank, country of training, and level of knowledge about t-PA in stroke as independent variables (Table 3).

All statistical tests were considered significant at p < 0.05. Data were analyzed using the statistical program SPSS (version 20.0).

## 3. Results

Out of 450 emergency physicians, 122 from ten hospitals in Riyadh participated in the survey with a 27% response rate. The majority of participants were men (78%) and Saudi nationals (93%), with a mean age of 40 ± 8 years. Half were board certified in emergency medicine. Nearly half were emergency residents in training, 36% were consultants, and the remaining were either associate consultants, assistant consultants, or staff physicians.

Regarding years of experience, 58.1% had 1–5 years of experience, 29.5% had 5–10 years, and 12.2% had more than 10 years of experience. Nearly half (49%) work for one large tertiary care hospital with an established stroke center. Two-thirds had an acute stroke team at their hospital with an ER-written protocol/care pathway for acute stroke management. The demographics and general characteristics of participants are shown in Table 1.

Table 2 shows the attitude of emergency physicians toward t-PA. Only 28.7% correctly classified the evidence as strong (high level of evidence), while the remaining considered the evidence to be weak (15.6%) or controversial (45.9) or were uncertain (9.8%). Furthermore, 45.9% recommend against its use due to lack of proven efficacy (37.5%), risk of hemorrhagic complications (30.3%), lack of stroke expertise (25%), and medicolegal liability (7.1%). When the stroke expertise is lacking, 35% recommended the training of ER physicians, and 24% suggested establishing telestroke to administer t-PA. In regard to the willingness of participants to enroll in training to administer IV t-PA for acute ischemic stroke, 52% were willing, while the rest were unwilling (32%) or uncertain (16.4%). Reasons for being unwilling included the decision to administer IV t-PA being too complex and needing specialized stroke expertise (24%), being uncomfortable to take the risk of thrombolysis (15%), medicolegal liability issues (11%), considering it a demanding service, and having no time to provide such service (13%). We also found that more than half (52%) of the participants are willing to administer IV t-PA for ischemic stroke in collaboration with remote stroke neurology consultation if telestroke is implemented. Multivariable logistic regression analysis did not show an association between any of the emergency physicians' characteristics and the knowledge toward recomending use of t-PA for ischemic stroke within the first 4.5 hours of symptoms onset.

## 4. Discussion

It has been more than two decades since the publication of the National Institute of Neurological Disorders and Stroke (NINDS) clinical trial that demonstrated a clear reduction in disability with intravenous t-PA in acute ischemic stroke within 0 to 3 hours of stroke onset [9]. Despite the American Food and Drug administration approval of the IV t-PA for stroke within 3 hours of symptoms onset in 1996, dissenting opinion and an attitude of antagonism toward t-PA among some medical groups continued. The concern raised was about the high risk of intracranial bleeding, potential effect of imbalance in baseline stroke severity between the two treatment groups in NINDS trial favoring the t-PA group, and the generalizability of the study [10]. However, the concern of imbalance in baseline stroke severity was refuted in a subsequent comprehensive and independent reanalysis of data [11]. The subsequent trials, published between 1995 and 2002, were European Cooperative Acute Stroke Study (ECASS), ECASS II, and Alteplase Thrombolysis for Acute Noninterventional Therapy in Ischemic Stroke (ATLANTIS). A/B were all negative which raised further skepticism [12–15]. However, these studies tested the efficacy of IV t-PA from 0 to 6 hours of symptoms onset and the meta-analysis of these trails demonstrated benefit of t-PA in the first 4.5 hours of onset [16]. Later, the European regulators requested to conduct the ECASS III which confirmed the benefit between 3.0 and 4.5 hours [17]. Parallel to ECASS III, The IST3 trial was based on the uncertainty principle, that is, patients enrolled when doctor is unsure whether t-PA would be of benefit. IST3 clearly demonstrated that, even in these subgroups of patients with a perceived marginal risk-benefit, t-PA within 3 hours of stroke onset decreases disability [18]. Later, the individual patient meta-analysis published in 2014 that included 6756 patients clearly demonstrated the benefit of t-PA within 4.5 hours. So it is clear that some of the skepticism about the benefit of t-PA in stroke was not unreasonable and the evidence accumulated over time.

Our study found that a large percentage of emergency physicians hold a negative attitude toward t-PA use in acute stroke. Almost half of the study participants recommend against its use, see it as ineffective, and consider it controversial. The main reasons for emergency physicians to recommend against t-PA use were lack of efficacy and perceived risk of hemorrhagic complications. Neither age, years of experience, job titles, qualification, nor hospital type influenced endorsement. Two-thirds of the participants work in tertiary care centers, where they have stroke teams and written stroke care protocols and clinical pathways; nevertheless, that did not influence their attitude. Similarly, knowledge of the stroke literature and guidelines did not change their perception of t-PA. Similar to our results, a survey of emergency physicians in Arizona and Missouri showed that only 48% of the participants believe that IV t-PA is an effective treatment for acute ischemic stroke [19]. In the 2009 survey by The American College of Emergency Physicians, 2,600 of its active members were randomly selected. Forty percent of physicians would not use t-PA due to the risk of intracerebral hemorrhage (65%) and the relative lack of benefit (23%) [20].

These contrarian viewpoints of Saudi emergency physicians are likely related to multiple factors. First, it might be just a part of the widespread dispute on the effectiveness of t-PA in the emergency medicine community worldwide. The skepticism and arguments have not settled since the Food and drug Administration's approval of t-PA in 1996. For example, the Canadian Association of Emergency Physicians Position Statement on Acute Ischemic Stroke recommends against the use of t-PA outside of the research setting in 2001 [21]. After 14 years of debate, the same association recommended the use of t-PA within the first 3 h of onset (strong recommendation, high level of evidence) but recommended against its use beyond 3 h, except in specialized stroke centers with advanced imaging capabilities or as part of a research protocol, despite increasing evidence supporting its effectiveness [22]. In the United States, it took the American College of Emergency Physicians a while to settle this dispute [12]. In fact, many publications in the field of emergency medicine advocate against the use of t-PA [23–25]. Second, poor knowledge of updated stroke management guidelines could have contributed to this. This was not evident in our subanalysis, but this could be due to the small sample size. Third, the acute stroke management pathway in some centers bypasses the emergency physicians where EMS personnel or triage desk nurses call the stroke team immediately, which leads to disengagement. Furthermore, exclusion or no participation of emergency physicians in stroke leadership, improvement initiatives, research, and collaboration might have contributed [8].

## 5. Conclusion and Recommendation

The emergency physicians are key in stroke care. Their negative view and attitude toward stroke thrombolysis might have a deleterious effect on the effective and timely administration of t-PA for stroke. It is crucial that Saudi emergency physicians be involved, educated, and engaged in stroke care, particularly during the health care transformation in Saudi Arabia. Many emergency physicians are policymakers; they lead the EMS and prehospital stroke care. They are front liners in the acute stroke management. The stroke and neurology community should collaborate more and involve emergency physicians in stroke care planning and implementation and engage them in all stroke initiatives across the country.

## Figures and Tables

**Table 1:** The general characteristics of the participating physicians.

**Variable**	**Frequency**	**Percentage**
Gender		
Male	96	78.6

Age		
< 30	51	41.8
30–40	60	49.1
> 41	11	9.0

Nationality		
Saudi	114	93.4
Non-Saudi	8	6.5

Qualification (board certification in emergency)		
Yes	61	50.0
No	61	50.0

Job title (Rank)		
Consultant	44	36.0
Resident in training	62	50.8
Others	16	13.1

Country of board certification (N=79)		
Saudi Arabia	42	53.1
North America	5	6.3
Still not board certified	27	34.1
Other	5	6.3

Years of experience		
1–5	71	58.1
5-10	36	29.5
> 10	15	12.2

Average strokes seen per week		
< 1	10	8.1
1–5	97	79.5
6–10	15	12.2

Type of hospital		
Tertiary care	80	65.5
Secondary care	42	34.4

Do you have an acute stroke team?		
Yes	83	68.0
No	33	27.0
I don't know	6	4.9

written protocol/ care pathway of acute stroke management?		
Yes	80	65.5
No	26	21.3
I don't know	16	13.1

**Table 2:** Physicians' knowledge and attitude toward t-PA.

**Questions **	**Response **	**Frequency**	**Percentage**
How would you rate your knowledge about t-PA use in ischemic stroke?	Well updated about most recent literature and guidelines	65	53.2
	General knowledge but acceptable	50	40.9
	Poor knowledge	7	5.7

Do you think t-PA is an effective treatment for stroke within 4.5 hours of onset?	Yes	70	57.4
	No	35	28.7
	I don't know	17	13.9

Do you consider t-PA a standard of care for ischemic stroke within 4.5 hours from onset in eligible patient?	Yes	57	46.7
	No	50	41.0
	I don't know	15	12.3

How would you grade the level of evidence for the use t-PA in ischemic stroke within 4.5 hours of onset?	Strong (high level)	35	28.7
	Weak (low level)	19	15.6
	Controversial	56	45.9
	I don't know	12	9.8

Do you recommend t-PA in acute ischemic stroke within 4.5 hours of onset for eligible patients?	Yes	66	54.1
	No	50	41
	Uncertain	6	4.9

If you don't recommend t-PA use in stroke, what would be the main reason? (n=56)	Risk of hemorrhage	17	30.3
	lack of benefit	21	37.5
	Medico-legal liability	4	7.1
	Lack of stroke expertise	14	25.0

In the absence of stroke expertise, what do you recommend?	No t-PA should be offered	42	34.4
	Train emergency physicians to give t-PA.	43	35.2
	Train internists to give t-PA	7	5.7
	Establish telestroke.	30	24.6

When needed, would you be willing to be enrolled in training to administer t-PA for stroke (similar to t-PA for myocardial infarction)?	Yes	63	51.6
	No	39	32.0
	Uncertain	20	16.4

If telestroke is implemented, would you be willing to administer IV t-PA for ischemic stroke in collaboration with remote stroke neurology consultation?	Yes	64	52.5
	No	31	25.4
	Uncertain	27	22.1

**Table 3:** The frequency, odds ratio, and adjusted odds ratios of emergency physicians who recommended use of t-PA in stroke.

Characteristics	Frequency % *∗*	Odds Ratio (95% CI) †	Adjusted Odds Ratio (95% CI) ‡
Total	54.1		

Age, y			
< 30	64.7	2.1 (1.01- 4.4)	2.7 (0.8 – 9.5)
≥ 30	46.4		

Gender			
Male	56.2	1.5 (0.6 – 3.5)	0.46 (0.12 – 1.2)
Female	46.1		

Nationality			
Saudis	54.3	1.1 (0.28 – 5.0)	1.02 (0.18 – 5.6)
Non-Saudis	50.0		

Years of experience, y			
< 10	54.2	0.96 (0.32 – 2.8)	1.12 0.29 – 4.3)
≥ 10	53.3		

Working in hospital designated as stroke center			
Yes	60.0	1.3 (0.43 – 3.95)	0.60 (0.17 – 2.10)
No	53.2		

Board certification in emergency medicine			
Yes	47.5	0.58 (0.28 – 1.20)	1.39 (0.40 – 4.81)
No	60.6		

Job Rank			
Consultant	52.2	0.89 (0.42 – 1.87)	0.51 (0.15 – 1.75)
Others	55.1		

Country of training			
Saudi Arabia	50.0	0.70 (0.34 – 1.44)	0.84 (0.31 – 2.25)
Others	58.6		

Level of knowledge about t-PA in stroke up to 4.5 hours of onset			
Well updated about recent literature and guidelines	42.8	0.49 (0.22 – 0.97)	1.8 (0.84 – 4.22)
General but acceptable or poor knowledge	61.6		

*∗*Percentage of emergency physicians who responded “yes” for the question, “Do you recommend t-PA in acute ischemic stroke within 4.5 hours of onset for eligible patients?”

†Estimated by Mantel-Haenszel method.

‡Results of multiple logistic regression with emergency physicians recommending t-PA use as the dependent variable and age, gender, nationality, years of experience, working in hospital with stroke center, board certification, job rank, country of training, and level of knowledge about t-PA in stroke as independent variables.

## Data Availability

The data used to support the findings of this study are available from the corresponding author upon request.

## Additional Points

*Limitations*. The major limitation is low response rate. However, 27% is somewhat acceptable for an Internet-based survey. We do not have information on the nonresponders. It is possible that those who did not respond may possess certain attitude which introduces bias. Furthermore, the study is limited to the city of Riyadh, and generalization to the whole country might not be valid. Also, the statistical power is limited due to small sample size.
